# The Reformed Public House

**Published:** 1924-01

**Authors:** A. P. Grubb

**Affiliations:** 9, John Street, W.C. 2


### The Reformed Public House.

SIR,—Mr. Simons's plea for a free hand for the brewers to reconstruct public-houses after a reformed model is specious but unconvincing. One of the largest brewers in the country, Mr. Waters Butler, admitted to an interviewer some time ago that the brewers themselves were powerless to effect any real reforms on a large scale in the conditions of the retail sale of drink. The truth is that since the tied-house system was established, the old English hostelry, noted for its hospitality and good cheer, has been superseded by the pothouse and gin-palace of the Limited Liability Companies, whose ideal licensed house is one capable of distributing the greatest quantity of alcohol in the smallest possible space. The Public House Improvement Bill, for which Mr. Simons desires Government facilities, would rivet the tied-house system more firmly to the neck of the community, and fatally weaken the present inadequate powers of controlling licensing matters in the hands of the public.

The only line of advance which promises any real advance in the direction of the reformed public-house is Local Option, with an option for the application of direct disinterested management.

A. P. GRUBB.

9, John Street, W.C. 2.

